# Let Nature Take Its Course: Cultural Adaptation and Pilot Test of Taoist Cognitive Therapy for Chinese American Immigrants With Generalized Anxiety Disorder

**DOI:** 10.3389/fpsyg.2020.547852

**Published:** 2020-09-17

**Authors:** Doris F. Chang, Nancy Ng, Teddy Chen, Tiffany Hung, Iris Yi Miao, Yuping Cao, Yalin Zhang

**Affiliations:** ^1^Silver School of Social Work, New York University, New York, NY, United States; ^2^New York City Police Department, New York, NY, United States; ^3^Charles B. Wang Community Health Center, New York, NY, United States; ^4^Department of Psychology, New School for Social Research, New York, NY, United States; ^5^Mental Health Institute of Second Xiangya Hospital, Central South University, Changsha, China

**Keywords:** culturally-adapted therapy, Chinese, immigrants, Taoism, anxiety, acculturation, Asian American

## Abstract

This report describes initial results from a multi-stage project to manualize and adapt an indigenous therapy, Chinese Taoist Cognitive Psychotherapy (CTCP), for dissemination in the United States context. Study aims were to (a) integrate cultural adaptation and implementation science frameworks to manualize and adapt the original intervention and (b) explore the feasibility, acceptability, and effectiveness of the modified intervention, renamed Taoist Cognitive Therapy (TCT), in a sample of Chinese immigrants with generalized anxiety disorder (GAD). Incorporating bottom-up and top-down adaptation approaches, we involved Chinese American clinician stakeholders and experts in Taoist philosophy, cognitive-behavioral therapy, and GAD to help identify cultural and contextual barriers and solutions to enhance treatment acceptability and implementation. Five treatment-seeking Chinese American immigrants (average age = 43.2 years) with a primary diagnosis of GAD completed 14–16 sessions of TCT. Two participants also had secondary diagnoses of major depressive disorder. Changes on primary measures of worry and anxiety were assessed for statistical and clinical significance using reliable change indices (RCIs; Jacobson and Truax, 1991) and comparisons to normative data. In this sample of patients with limited prior exposure to Taoism, results found evidence of feasibility and acceptability of the modified intervention, with strong endorsement of Taoist principles at termination. Statistically and clinically significant improvements in anxiety, worry, psychological inflexibility, and emotional avoidance were found only for the participants without comorbid depression. Results suggest that Taoist principles of acceptance and flexible adaptation to natural laws may be helpful to Chinese immigrants coping with anxiety. However, additional treatment modifications may be required to address the low self-efficacy and fatalism expressed among those with comorbid depression.

## Introduction

While most influential psychotherapeutic interventions have been developed in Western contexts and exported worldwide, the rise of mindfulness and other contemplative interventions has highlighted the psychological wisdom that may be found in principles and practices originating in non-Western cultures (Murguia and Díaz, 2015; Moodley et al., 2018). Building on this trend, this report describes initial results from a multi-stage project to manualize and adapt an indigenous therapy developed in China, Chinese Taoist Cognitive Psychotherapy (CTCP), for dissemination in the United States context.

CTCP was developed in the 1980s by two Chinese psychiatrists, Zhang Yalin and Yang Desen, to address the somatic concerns and existential worries described by their Chinese patients during the post-cultural revolution reform period (Zhang and Young, 1998; Young et al., 2008). Despite global improvements in standards of living, China’s turbulent transition from a socialist planned economy to a market economy produced large-scale social and economic dislocation (Sun and Ryder, 2016; Yang, 2017). Within the crowded hospital consulting rooms, patients described mounting anxiety and worries about the future as they struggled to navigate this shifting cultural landscape. Facing the inadequacy of standard therapeutic approaches, the clinicians drew on Taoist ideas of acceptance and flexible adaptation to natural laws to help patients cope with the unpredictable nature of modern Chinese life (Zhang and Young, 1998). Eventually they standardized their approach in the form of a Taoism-oriented cognitive behavioral therapy (CBT) that targeted patients’ distorted views of the world as predictable and controllable and promoted coping responses aligned with the Tao (Zhang et al., 2002; Young et al., 2008).

CTCP represents a radical departure from the Western therapeutic modalities that comprise the bulk of mental health interventions practiced in China in that it centers on local indigenous perspectives about the nature of the world. Viewed properly as a folk-influenced culturally-adapted intervention, rather than a religious or spiritual approach to treatment, CTCP teaches patients to apply tenets and values of philosophical Taoism, in particular, promoting communal (versus individual) well-being, tempering excess, cultivating humility and flexibility, and aligning with the laws of nature (Zhang et al., 2002).

### Comparing CTCP and Traditional CBTs

Similar to traditional CBT approaches, CTCP shares the view that psychological problems are based in part on faulty or maladaptive thinking patterns and that the goal is to replace unhelpful thinking and behavioral patterns with those grounded in a more accurate understanding of reality (Beck, 1976; Burns, 1980). However, the two approaches differ somewhat with regard to their philosophical views on the nature of reality and the explicit values that they associate with wellness and recovery. CBT emphasizes logic and reason, engaging the patient in Socratic questioning to uncover and correct cognitive distortions about the self, others, and the world. This process socializes the patient toward values of rationality and linear thinking, agency and action, reflecting the influence of Stoic philosophy on its developers (Beck et al., 1979). CTCP shares a focus on rationality and a recognition that we can control our thoughts, judgments, and actions, but not life’s outcomes. However, to better reflect Chinese cultural worldviews and values, it embeds a distinctly Taoist view of the world, one that is inherently more holistic, harmonious, and interdependent, and *explicitly* promotes a set of prescriptive philosophical values aimed at strengthening the individual’s alignment with the Tao, or the natural order of the universe.

In contrast to a Western cultural emphasis on individual mastery, agency, and control, Taoist philosophy asserts that the natural order of the universe is one of functional harmony. Problems arise when individuals seek to act in ways that violate or disrupt the balance and interdependence that naturally exists (Mitchell, 2006, ch. 2). As individuals realign themselves with this natural order – rationally appraising oneself and others, abolishing binary thinking (e.g., good/bad), cultivating humility and patience, and flexibly adapting to situations as they arise – they can live with greater ease.

Harmony and balance are sought in all things. Through learning and embodying key Taoist virtues and principles, the patient in CTCP is encouraged to (a) consider others’ needs alongside their own (*Compassion*: “Benefit without harm/Act but do not compete”), (b) regulate their excessive impulses and desires (*Moderation*: “Limit possessions and moderate desire/Know when it is enough, know when to stop”), (c) be flexible and humble in relationships (*Humility*: “Know harmony and be modest/The soft can overcome the tough”), and (d) seek balance and alignment with the Tao/nature [*Effortless Action*: “Be clear and still and rest in nonaction (*wuwei*)/Let nature take its course”]. Stories and proverbs are used to illustrate these teachings, which are presented in a series of rhyming couplets so as to be culturally familiar and easy to recall, accessible to patients who are semi-literate. Rather than using thought records to correct cognitive distortions, the patient learns to use these core Taoist principles to analyze situational stressors and relationships and practice new ways of responding (Chang et al., 2016).

While CTCP may be viewed as a departure from traditional Western therapeutic modalities, it is important to note that the developers of CBT were themselves influenced by Eastern philosophies of Buddhism and Taoism, as well as Western Stoic and Existentialist philosophical traditions (Murguia and Díaz, 2015). In other words, traditional CBT approaches, including Rational-Emotive Behavioral Therapy (REBT), contain the seeds of Taoist thinking. As Murguia and Díaz (2015) note, Albert Ellis acknowledged that his formulation of REBT was influenced by several Taoist ideas, including the rejection of categorizations as misrepresentations of what is in constant flux, and the view that through intention and practice, we can move toward a state of greater ease and flow (Ellis and Harper, 1997). In CTCP, these ideas are given center stage, alongside other Taoist philosophical ideas, to better reflect East Asian cultural values and worldviews.

### Applying CTCP With Chinese American Immigrants: Considering the Need for Cultural Adaptation

Studies conducted in China have found empirical support for CTCP as a treatment for a variety of conditions including anxiety (Zhang et al., 2002; Li and Chen, 2009), depression (Yang et al., 2005; Li et al., 2014; Liu and Yu, 2015), somatoform disorders (Cao et al., 2015), and stress-related health conditions (Zhu et al., 2005, 2012). In one study, 143 Chinese patients with generalized anxiety disorder (GAD) were randomized to CTCP, benzodiazapines (BZD), or combined CTCP and BZD (Zhang et al., 2002). By termination, the CTCP group showed significantly greater improvements in anxiety with the combination of CTCP and BZD produced the most rapid and enduring improvements. Despite growing evidence of its effectiveness, however, CTCP has never been disseminated outside China. The absence of a detailed treatment manual, rigorous training guidelines, questions about transportability of the model given the uniqueness of the Chinese treatment context, and lack of English-language materials has hindered dissemination outside of China.

In 2019, 27% of the more than 12 million Chinese living outside of China resided in the United States. Chinese immigrants represent the third largest group in the total United States foreign-born population, after immigrants from Mexico and India (Echeverria-Estrada and Batalova, 2020). Like their counterparts experiencing sweeping social changes in China, Chinese immigrants also face significant disruptions in family structures and routines, financial uncertainty, changing cultural norms, and the need to develop adaptive coping responses. Such acculturative stressors have been linked to mental disorder (Hwang and Ting, 2008), with anxiety being the most common manifestation of distress, affecting one in 10 Asian Americans across the lifespan (Hong et al., 2014). Yet, Asian Americans with mental disorders consistently underutilize mental health services compared to European Americans and other ethnic groups (Sue et al., 2012).

One key factor is the cultural mismatch between patients and providers, particularly with regard to explanatory models of illness and healing. Meta-analytic studies involving diverse samples show that culturally adapted psychotherapy is more effective than unadapted bona fide psychotherapy (Benish et al., 2011; Hall et al., 2016; Smith and Trimble, 2016). However, the great variability in intervention effect sizes has prompted examination of potential moderators, including study design characteristics, the specific nature of the cultural adaptations, and characteristics of the treatment population. Results indicate that the effects of cultural adaptations may be accounted for by the degree of cultural match between the intervention and patients’ explanatory models of illness and treatment goals and the use of indigenous frameworks and metaphors that reflect patients’ cultural worldviews (Cabassa and Baumann, 2013).

Tailoring the intervention to a specific cultural subgroup has also been associated with stronger treatment effects. Huey and Tilley (2018) examined treatments designed specifically for Asian Americans in 21 randomized trials (*n* = 6,377 participants) and found that those tailored for specific Asian subgroups (e.g., Chinese Americans) produced substantially larger effects (*d* = 1.10) compared to those without culture-specific tailoring. However, treatment effects varied by problem type, with only a small number that targeted anxiety disorders other than PTSD.

### The Present Study

To increase the availability of culturally-accessible services for Chinese immigrants, as well as other East Asian groups who share a similar cultural heritage, we launched a multi-phase international collaboration to manualize, enhance, and adapt CTCP for dissemination in the United States context. Models from the cultural adaptations and implementation science fields informed our approach to identifying cultural and contextual factors that may affect the relevance, acceptability, and success of the intervention (Bernal et al., 1995; Graham et al., 2006; Durlak and DuPre, 2008; Hwang, 2009). Cultural adaptation refers to the systematic modification of an existing treatment to improve its compatibility with the culture, language, and context of the client (Bernal et al., 2009). Numerous cultural adaptation frameworks have been developed to guide the evaluation of core elements that may require adaptation and to document the resulting adaptations for replication and dissemination purposes (Bernal et al., 2009). The field of implementation science seeks to promote evidence-based practices (EBP) in health care more generally by identifying the contextual factors and processes that lead to the success and failure of EBPs and developing approaches to enhance the update and integration of EBPs into routine practice (Nilsen, 2015). Implementation researchers have developed numerous conceptual frameworks to consider how characteristics of the EBP, the users/providers, the patients, context, and means of implementation may inform its success or failure. As noted by Cabassa and Baumann (2013), integrating advances in cultural adaptations and implementation science may help identify which types of cultural adaptations preserve the effectiveness of evidence-based interventions, while considering how the context that surrounds the intervention may also need to be adapted to accommodate and improve their acceptability and uptake.

Although CTCP was developed for Chinese populations, there are distinct differences in implementation context and approaches to treating anxiety that may affect the success of the intervention in the United States. For example, given the structured nature of CTCP – requiring specialized training on the part of the provider and regular visits with the patient – it is typically delivered by Chinese psychiatrists working in outpatient hospital settings rather than community clinics. In these profit-oriented institutions, mental health services are costly and less accessible to the average Chinese, such that the patients who typically receive psychotherapy in China today come from the middle to upper classes (Yang, 2017). This medical treatment context helps to contextualize why so many published studies on CTCP have targeted depression and anxiety in patients with chronic medical conditions such as diabetes, stroke, or coronary heart disease, and frequently pair CTCP with pharmacologic interventions.

In contrast, we identified the most suitable United States implementation context for this intervention to be Asian ethnic-specific service agencies that aim to deliver culturally-responsive care for Chinese immigrant populations. These ethnic-specific clinics are typically embedded within communities to improve access and engagement, with therapy services more commonly delivered by social workers and psychologists. Chinese immigrants also have a lower median household income and are less likely to have attended college compared to United States-born Chinese Americans (Pew Research Center, 2017). Depending on how long they have been in the United States and age of immigration, they may identify less with Chinese cultural traditions, including Confucianism, Buddhism, and Taoism, than their counterparts in China. Finally, training of social workers and psychologists in the United States tends to focus on social and intrapsychic processes with less attention to the body. As Yang (2017, p. 111) observed, “Whereas Western psychotherapy addresses feelings in a bounded, masterful individualistic self that arises from intrapsychic processes, many Chinese counselors (in China) pay greater attention to the body-heart nexus… that is, the body acts both as the bridge reaching hearts and as a force transducing other social forces.”

Yang’s observations reflect more general findings from the field of cultural psychology documenting how cultures vary systematically with regard to constructions of the self. Whereas most Western cultures emphasize an independent schema of self, viewing intrapsychic processes (thoughts, feelings, and actions) as its primary referent, East Asian cultures tend to emphasize an interdependent schema of self, in which the self is seen as connected to and defined by one’s relationships, roles, and responsibilities to one’s ingroup (Markus and Kitayama, 1991, 2010). Within this latter formulation, the boundaries surrounding the self are more fluid, such that expressions of selfhood may be more dynamic and more inclusive, incorporating mind, body, spirit, and society. Ryder et al. (2002) reviewed findings regarding the centrality of somatic presentations of mental illness in China, suggesting that this pattern may be in response to a number of cultural forces: the pressure to suppress strong emotions to maintain harmony within the group, the stigma of mental illness, and theories of Chinese medicine that integrate psychological, physical, and social factors in a more holistic understanding of self and health. With regard to our current study, it is unclear how the particular context of service delivery, patient characteristics, and professional models of health and healing may influence the delivery and receipt of CTCP and what modifications may be required for successful implementation with Chinese American immigrants receiving community-based services.

Our approach to cultural adaptation was guided by both theory-driven, or “top down” methods as well as community-participatory, or “bottom-up” methods (Bernal et al., 1995; Hwang, 2009). The theory-driven ecological validity model of Bernal et al. (1995) highlighted eight dimensions potentially requiring adaptation: language, persons, metaphors, content, concepts, goals, methods, and context. However, to guide our decision-making around these components, we engaged diverse stakeholder groups, including experts in philosophical Taoism and contemplative approaches to the treatment of anxiety, as well as bilingual and bicultural clinicians and Asian American members of the research team to help identify potential content and process barriers to implementation, as more recent models recommend (Movsisyan et al., 2019). Although it was published after the adaptation phase of this project, the Formative Method for Adapting Psychotherapy (FMAP) of (Hwang, 2009) outlines a similar community-based approach to cultural adaptation consisting of five phases: (a) generating knowledge and collaborating with stakeholders, (b) integrating generated information with theory and empirical and clinical knowledge, (c) reviewing the initial culturally adapted clinical intervention with stakeholders and revising the culturally adapted intervention, (d) testing the culturally adapted intervention, and (e) finalizing the culturally adapted intervention.

In a small feasibility study, the adapted intervention, Taoist Cognitive Therapy (TCT) was piloted with five Chinese American immigrants with GAD. We focused on GAD given its chronicity and prevalence in the population, comorbidity with other anxiety and mood disorders, and high levels of functional impairment. In this report, we describe our manualization and adaptation procedures and the feasibility, acceptability, and preliminary effectiveness of the adapted intervention, TCT.

## Materials and Methods

### Adaptation and Development of TCT

Table 1 outlines the phases of the manualization, adaptation, and revision process. Although the treatment developers had developed a brief outline of the CTCP intervention used for training purposes in China, it lacked sufficient detail to train American novices with minimal cultural exposure to Taoism. To obtain a more complete description of the original intervention, we began by recruiting two patients (in Beijing and Shanghai), who agreed to allow their entire CTCP treatment to be video-recorded for research and training purposes. Recordings were carefully reviewed with our Chinese collaborators (including Y.Z., one of the original developers of CTCP) to identify core treatment components, therapeutic techniques, and presentation strategies. This formed the basis of an initial English-language draft of the treatment manual (Phase 1).

**Table 1:** Procedures for manualizing and developing Taoist Cognitive Therapy (TCT), an adaptation of Chinese Taoist Cognitive Psychotherapy (CTCP) for application to the United States context.

Phase 1: Description of CTCP framework and procedures. Didactic training in CTCP by Dr. Zhang Yalin. Collection of video recordings and case material from actual cases of CTCP treatment in China. Review and analysis of session videos and discussion with Dr. Zhang and the Chinese research team.	→	Complete draft of CTCP manual
Phase 2: Consultation with Taoist and clinical experts. Consultation with Taoist expert to clarify interpretation and translation of core Taoist concepts. Consultation with experts in generalized anxiety disorder (GAD) and acceptance-based and behavioral therapies on the CTCP conceptualization of GAD and its treatment components. Ongoing discussion with collaborators in China and the United States research team, consisting of immigrant Chinese students led by the primary investigator, a Chinese American clinical psychologist with experience treating Chinese immigrant patients with anxiety. Produce an initial draft of the TCT intervention based on theory-driven modifications of CTCP.	→	Initial draft of TCT manual (TCT v1)
Phase 3: Review by Clinician Stakeholders and Revision. Presentation of TCT v1 to 16 Chinese bicultural/bilingual clinicians and clinic administrators and discussion. Description of prototypical patient profiles. Identification of barriers to implementation related to patient and clinician characteristics, usual care practices clinical structures, and exploration of possible solutions. Modification of treatment in response to stakeholder input.	→	Second draft of TCT manual (TCT v2)
Phase 4: Initial testing of TCT v2 – open trial. Assess feasibility, acceptability, and treatment outcomes with five Chinese patients with GAD. Interview stakeholders (therapists) to explore challenges in implementing TCT. Revision of TCT based on user experience and patient response.	→	Third draft of TCT manual (TCT v3)

In Phase 2, we consulted with an internationally known scholar of Taoism to ensure that descriptions and interpretations of the core Taoist teachings in the treatment manual were accurate. Experts in mindfulness‐ and acceptance-based approaches to treating GAD were consulted to clarify unique features of CTCP and strengthen the theoretical framework. Guided by the ecological validity model of Bernal et al. (1995), initial modifications were made in the domains of *content* (development of training modules on Taoist principles for Chinese-American clinicians and patients; elaborating the theoretical framework), *goals* (increase patient engagement due to cultural relevance of Taoist values and worldviews), *methods* (development of a treatment manual, patient handouts, and thought records), and *context* (consideration of changes in contexts and their effects, e.g., acculturative stressors, immigrant status, and United States health care setting). With regard to *methods* in particular, assessment materials used in China were evaluated and replaced with tools used more widely used in the United States clinical context. Patient handouts, which were not used in CTCP, were created as tools for instruction and application of core concepts, in line with standard CBT practices.

The training modules and revised training manual were then presented to a group of 16 bilingual and bicultural Chinese American clinicians, including the director of an Asian-specific mental health program, who provided feedback on areas in need of clarification as well as barriers and considerations for implementing the intervention with Chinese immigrant patients (Graham et al., 2006; Durlak and DuPre, 2008; Hwang, 2009).

The clinician stakeholders’ feedback may be organized into two main themes. First, they expressed concerns that popular views of *Taoism as the Domain of Priests and Scholars* would reduce the approachability of the intervention among the Chinese lay public. The clinicians also questioned their own competence in delivering a Taoism-focused intervention given their lack of formal training in Taoist philosophy. After clarifying that the intervention was grounded in simple, practical precepts of Taoist philosophy, which are reflected in numerous Chinese folk stories and proverbs, they were more receptive to the possibility that with training they could faithfully deliver the intervention. Their feedback led to two key modifications to the treatment. We adopted the stakeholders’ suggestion to initially present the intervention to patients as a “therapy grounded in traditional Chinese wisdom” in order to increase patient engagement. Specific references to Taoism now do not occur until Session 4, just before the clinician begins teaching the eight Taoist principles. We also enlisted the help of our expert consultant on Taoism to develop an “Introduction to Taoism” training module for the clinicians to provide relevant background information and instructional guidance in the eight principles.

Second, clinician stakeholders raised concerns about *Cultural Differences in Views of the Patient-Provider Relationship*. In viewing the videotapes of the Chinese training cases collected in Phase 1, we observed that the patient-provider relationship dynamic was that of expert teacher and student. The clinician was clearly the authority figure, as exemplified in their wearing of a white doctor coat and their adoption of a didactic tone, prescribing Taoist principles as the antidote to the patient’s problems and concerns. The hierarchical quality of the therapeutic relationship has been previously described in studies of clinical care in China (Yang, 2011) and reflects the cultural organization of social relationships according to status or occupation.

However, the Chinese American clinician stakeholders expressed discomfort adopting an authoritative stance with their patients. Reflecting the more egalitarian, patient-centered values of therapeutic practice in the United States, the clinicians stated their preference for a more equal distribution of decision-making power and cultivation of a warm and trusting relationship with their patients. They further indicated that they would be more likely to adopt a more Socratic approach to teaching the eight principles in order to stimulate the patient’s independent thinking and self-inquiry regarding their values and beliefs about the world, consistent with standard cognitive-behavioral approaches.

Differences in how clinicians negotiate their professional roles and relationship to the patient may reflect fundamental differences in cultural values (Schwartz, 1999, 2006; Hofstede, 2001). Notably, Schwartz’ theory of seven cultural value orientations on which all societies may be compared includes the polar dimensions of *hierarchy* vs. *egalitarianism* (Schwartz, 1999, 2006). These values are both described as responding to the need for societies to ensure that members will behave responsibly toward one another to preserve the social fabric. Cultures that value *hierarchy* rely on hierarchical systems of prescribed roles to ensure socially responsible behavior and socialize members to comply with the duties and responsibilities of their roles. In contrast, cultures ranking high on *egalitarianism* emphasize the shared human interests that unite members, socializing members to view each other as moral equals and voluntarily commit to concerning themselves with the well-being of others. Comparative research provides evidence that participants in China rank particularly high on relational hierarchy even relative to other East Asian groups (Zhang et al., 2005) and low on egalitarianism, whereas participants in the United States rank moderately high on both dimensions (Schwartz, 2006). While we did not formally alter the intervention to accommodate United States clinicians’ more egalitarian relational style, we wondered about the treatment implications of these differences in clinicians’ relational stance and authority in China and the United States. As a result, we decided to explore these issues in the post-treatment debriefing interviews with patients.

In summary, feedback elicited in Phase 2 informed subsequent revisions of the treatment manual, training protocols, and fidelity measure (Phase 3). The final protocol, which we refer to as TCT, updates and builds on CTCP by elaborating its conceptual framework, modifying and updating its assessment tools and procedures, providing extensive training protocols on Taoist concepts for clinicians and patients with little formal exposure to Taoism, and including a set of patient handouts and modified thought records to promote learning and application of concepts.

### Sample and Recruitment

#### Participants

To pilot test the adapted intervention, five new treatment-seeking Chinese immigrants (four women and one man) with a primary diagnosis of GAD were recruited in an open-trial conducted at an Asian ethnic-specific community health center. Intake clinicians were notified about the study inclusion criteria and asked to refer any patients who may be interested and eligible to participate. Exclusion criteria included suicidal ideation, psychosis, substance abuse, mental retardation, or organic brain damage.

Table 2 presents diagnosis and chief complaints for each case (all names are pseudonyms). Average age was 43.2 (*SD* = 11.5). All had voluntarily immigrated from Mainland China, Hong Kong, or Taiwan. Two identified as Fujianese, one Toisanese, one Hong Kong Chinese, and one identified as Taiwanese. Years in the United States ranged from 9 to 37 (*M* = 19.0, *SD* = 11.0). Two reported speaking English “somewhat well,” and three reported speaking “not much” English. All identified as heterosexual. Four were married and one was separated. Only one had graduated from college, and four were employed doing administrative or other semi-skilled work (three full-time and one part-time). One was identified as Buddhist; the other four reported no religious affiliation. Only one reported “a little bit” of knowledge of Taoism; the rest reported “no knowledge.” See Chang et al. (2016) for a case study of Mrs. Liu.

**Table 2:** Case description of the five participants and changes observed.

Case	Description	Changes observed
Mrs. Liu	GAD and PTSD (in remission)	“My level of anxiety is not that intense anymore. My thoughts were very extreme. I will not think of the positive aspect. I would think about the worst scenarios and thought all the bad things would happen to me. But now, I will not think of these things. I will think about that a lot of people are not as lucky as I am, I am very lucky. My thoughts have changed.”
Mrs. Chan	GAD and major depression	“It is good, I feel an improvement. I feel like my thoughts are changing and as a person I am more free and open. I learned new ways of thinking. I try not to have as much negative thoughts. I tell myself 順其自然 (Let nature take its course). Do not think too much. 清靜無為 (Be clear and still and rest in *wuwei*/nonaction). I use these principles the most. Still, even with little things, I get upset or sad. It is still hard to fully let go.”
Mrs. Huang	GAD	“I am less tardy and tired. I am able to go to work on time, take care of my daughter before school. Before treatment, I would feel exhausted and would not have the energy to do anything. Now I am able to get up and go to work on time, able to relax myself, and sleep better. I am not 100% better but I am 60–70% better. I also used to afraid to ride the subway, scared that I would fall and would need to grab hold of something.”
Ms. Xiao	GAD and major depression	“Not so much, I think it might be me. The therapist would ask me to do things that were supposed to help me, but I just did not do it, I did not “follow up.” So then of course, the result was not good. It might be that the treatment would help others. I wanted to give it a try but at the end, I felt like it did not help me that much. I did not see any changes to my symptoms because the causes of the problem (her finances and living situation) were not resolved.”
Mr. Lin	GAD	“My mood has improved and my attitude toward life as well. I find this program really helpful for me to think through things. The way I think has changed a bit. I used to worry a lot about my physical health. I have (chronic health condition) and often take medication. I always worry about this. But I realized that it is useless to worry so much. There is nothing I can do about it. All I can do is what I can do, I cannot really change that much.”

The Structured Clinical Interview for DSM-IV-Axis I Disorders-Patient Edition (SCID-I/P), Chinese version (First et al., 2002; Phillips et al., 2009) was used to confirm lifetime and current DSM-IV Axis I diagnoses. Participants who had comorbid anxiety or mood disorders were eligible only if they had a current primary diagnosis of GAD. Two patients (Ms. Chan and Ms. Xiao) had secondary diagnoses of major depression. Four patients were concurrently receiving pharmacotherapy, primarily for sleep problems and depressed mood.

#### Therapists

One male and one female licensed clinical social worker working at the same Asian ethnic-specific community health center delivered the intervention to 2 and 3 patients, respectively. The female social worker, identified as Chinese American, was born in the United States and had 4 years of clinical experience. She identified as Christian. The male social worker was an immigrant from Taiwan who was educated in the United States and had 28 years of clinical experience. Both reported fluency in both English and Mandarin and had limited prior knowledge of Taoism.

### Pilot Study Procedures

Participants received 14–16 weeks of individual therapy at no cost, and $85 for completing study assessments. Procedures were approved by the review boards of the university and collaborating clinic. Primary measures assessing target symptoms of worry and anxiety were administered at five time points [baseline, session 4, session 8, termination, and at a 4-month follow-up (4mFU)]. Secondary measures assessing depression, cognitive inflexibility, and social/interpersonal functioning were administered at baseline, termination, and 4mFU. Treatment credibility and expectancies were assessed after the second session. Assessments were administered in Mandarin using published translations. When translations were not available, measures were translated using established procedures (Brislin, 1980). Separate face-to-face individual debriefing interviews were conducted with each patient and clinician following termination.

### Measures

#### Feasibility

Feasibility was assessed by the ability to identify and recruit eligible patients and retain participants through a complete course of treatment.

#### Acceptability

The *Credibility/Expectancy Questionnaire* (CEQ; Devilly and Borkovec, 2000) was used to assess perceptions of the credibility of TCT and expectancies for change (score range 1–3). To assess endorsement of the Taoist principles that form the basis of TCT, the nine-item *Taoist Values Inventory* (TVI) was created for this study (score range 0 = not important at all to 7 = extremely important for promoting health and well-being). High internal consistency (*α* = 0.86) of the TVI was found in a sample of 121 Chinese American immigrants (Miao et al., 2016).

#### Preliminary Effectiveness

Statistically reliable and clinically significant changes were assessed on the following primary measures: the *Chinese-Penn State Worry Questionnaire* (Ch-PSWQ; Meyer et al., 1990; Zhong et al., 2009) assesses the generality, excessiveness, and uncontrollability dimensions of pathological worry. The *State–Trait Inventory for Cognitive and Somatic Anxiety-Trait Version* (STICSA-Trait; Grös et al., 2007) measures trait cognitive and somatic anxiety. Secondary measures included the *Chinese Patient Health Questionnaire* (PHQ-9; Kroenke and Spitzer, 2002; Chen et al., 2006), the *Action and Acceptance Questionnaire-II* (AAQ-II; Bond et al., 2011), which assesses psychological inflexibility and experiential avoidance, and the Interpersonal Relations (IR) and Social Role (SR) subscales of the *Outcome Questionnaire-45* (OQ-45.2; Boswell et al., 2013).

#### Debriefing Interview

After completion of the treatment, individual semi-structured debriefing interviews were conducted with each patient and clinician to explore their experiences of the treatment and the therapeutic relationship, solicit feedback regarding key treatment components, and recommendations for further improving the treatment.

### Treatment and Training

TCT was administered using the final manualized protocol (Chang et al., 2016, Unpublished). Stage 1 (Orientation and Assessment) assesses current difficulties, values, and coping strategies, introduces self-monitoring skills (thought records), and orients the patient to the TCT approach. Stage 2 (Taoist Doctrine Instruction and Application) teaches the core Taoist principles and assists the patient in applying them to situations that elicit worry and maladaptive coping. The eight core principles are presented in four couplets as follows: (1) Benefit without harm/Act but do not compete (利而不害,為而不爭), (2) Limit possessions and moderate desire/Know when it is enough, know when to stop (少私寡欲,知足知止), (3) Know harmony and be modest/The soft can overcome the tough (知和處下,以柔勝剛), and (4) Be clear and still and rest in nonaction (*wuwei*)/Let nature take its course (清靜無為,順其自然). The patient learns to use these principles to analyze situational stressors and sources of worry and anxiety using thought records designed for this purpose.

For example, in treating a patient who feels anxious and stressed due to a climate of competition with colleagues in the workplace, the therapist guides her to explore the implication of relevant Taoist principles including, “Act but do not Compete” (為而不爭). A handout provided to the patient discusses this principle as follows: “Defining yourself in relation to others can create harmful feelings of envy, shame, pride, or complacency. In striving to achieve your goals, do your best according to your own capacity. Accurately appraise your abilities, talents, and resources, as well as your limits and weaknesses so that you can set a realistic goal” (Chang et al., 2016). To help the therapist explain each principle to the patient, the treatment manual includes numerous teaching examples drawn from core Taoist texts as well as contemporary life. To explain the meaning of “Act but do not Compete” for example, the therapist may offer a metaphor from nature: “A predator will pursue prey in order to survive and will compete with other predators for the same target. However, they do not feel resentment or self-pity that another predator has caught more food. Rather, large predators will pursue as large a prey as they feel they can catch, small predators will likewise select their prey based on their ability to successfully attain it” (Chang et al., 2013, p. 31). Through use of a thought record, the patient applies this principle to her situation and realizes that her fear of falling short has led her to be self-critical and engage in unreasonable self-comparisons with more senior colleagues. She sees how this has contributed to her anxiety, shame, and lack of motivation. The patient and the therapist then proceed to explore her abilities, talents, and resources to help her establish reasonable self-expectations and goals for her work.

Stage 3 (Internalization and Reinforcement) tailors the patient’s application of Taoist concepts to key anxiety-producing situations, and reinforces behavioral responses that are consistent with Taoist principles. Two final sessions focus on reviewing treatment gains and preparing the patient for termination. Additional sessions may be added if needed, for example to respond to emergent crises or to provide further explanation of core concepts.

Study therapists completed four 3-h training sessions led by the principal investigator, a Chinese American clinical psychologist trained in CTCP by its original developer (Y.Z.). As part of the training, an expert in Taoism delivered an “Introduction to Taoism” lecture and provided instruction in the core Taoist principles used in TCT. Therapists received individual weekly clinical supervision. Session audio recordings were reviewed by the research team using a fidelity checklist to monitor treatment adherence.

### Analysis Plan

Given the small sample size, results are descriptive in nature. Symptom severity scores on the Ch-PWSQ, STICSA-Trait, PHQ-9, AAQ-II, and the OQ-45.2 IR and SR subscales were compared to published norms. To determine whether changes on the primary outcome measures (Ch-PWSQ and STICSA-Trait) were statistically reliable as well as clinically significant, reliable change indices (RCIs; Jacobson and Truax, 1991) were computed at the individual patient level. First, the RCI was calculated by subtracting the post-treatment score (termination and 4mFU) from the pre-treatment score and dividing by the standard error of the difference. An RCI of >|1.96| suggests that the magnitude of change is statistically reliable (*p* < 0.05). Second, if statistically reliable change was found, the clinical significance of the change was determined by examining the post-treatment score to see whether it fell within the range of scores for normative samples.

## Results

### Feasibility

All participants who were eligible were successfully recruited, and 100% participants were retained through the course of the treatment. However, due to participants’ restrictive work schedules and family responsibilities, the treatment period ranged from 8 to 12 months, with most patients scheduling sessions every 2–3 weeks. After session 14, the participants were permitted to continue to return for periodic follow-up sessions if desired. While two did not return, the remaining had an average of an additional two sessions between the termination interview and the 4mFU.

### Acceptability

After being presented with the treatment model after session 2, participants’ mean rating of treatment credibility (CEQ) was 1.57 (*SD* = 0.55, range 0.63–1.9) and the mean expectancy rating was 1.77 (*SD* = 0.72, range 0.53–2.12), indicating moderate endorsement. Scores on the TVI averaged 6.22 out of 7 (*SD* = 0.66) at termination and 6.20 (*SD* = 0.30) at the 4mFU, indicating strong and stable endorsement of the core Taoist principles.

### Treatment Effectiveness

#### Baseline Assessment

Pre-treatment, all participants’ scores on the Ch-PWSQ exceeded the clinical cut-off (>45) for generalized anxiety (Behar et al., 2003). Scores on the STICSA-Trait also exceeded the clinical cut-off (>43) for a probable anxiety disorder (Van Dam et al., 2013). Three cases reported depressive symptoms (PHQ-9) in the moderately severe range (>15; Kroenke and Spitzer, 2002; Chen et al., 2006). On the AAQ-II, four participants endorsed clinically significant levels of psychological inflexibility and experiential avoidance (>24; Bond et al., 2011). Two and four participants reported clinically significant impairments in SR functioning and interpersonal functioning, respectively, at baseline (OQ-45.2 SR and IR subscales; Boswell et al., 2013).

#### Changes in Primary Outcomes

**Figure 1:**
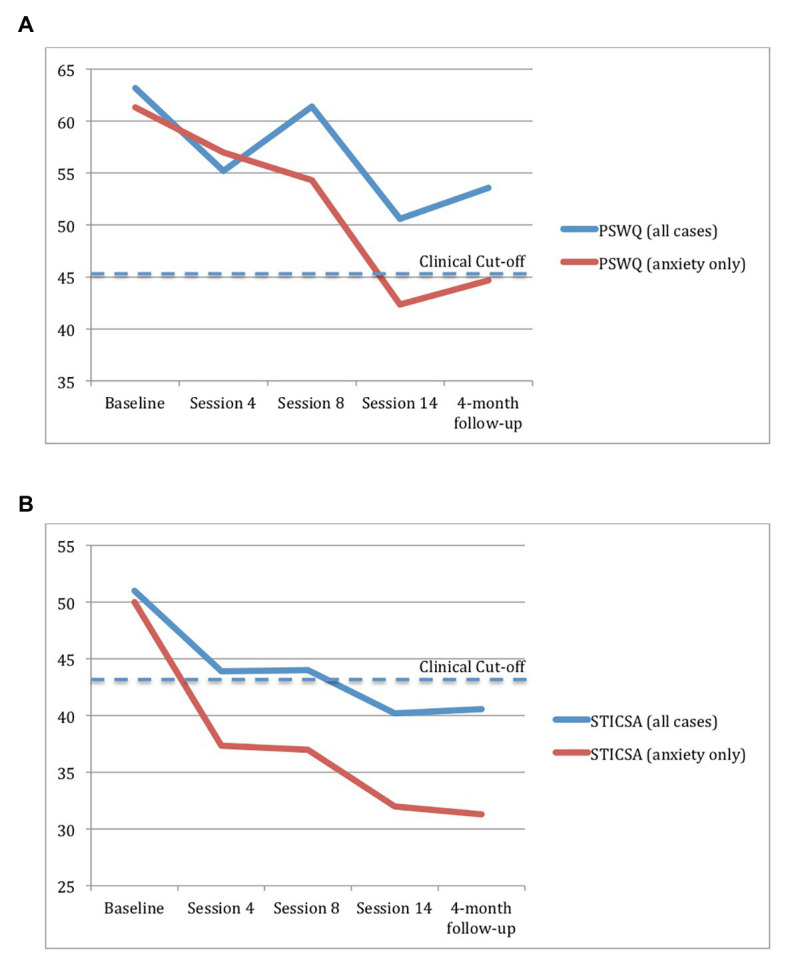
Self-reported worry **(A)** and anxiety **(B)** in the full sample (*N* = 5) and for participants with anxiety only (*n* = 3) at five time points. The cut-off score is the value above which a psychological disorder is likely. Cut points were obtained from Behar et al. (2003) for the Penn State Worry Questionnaire (PSWQ) and Van Dam et al. (2013) for the State-Trait Inventory for Cognitive and Somatic Anxiety-Trait Version (STICSA).

Figure 1 summarizes changes in the target symptoms of worry and anxiety over the course of treatment and at the 4mFU. Statistically and clinically significant improvements were observed in the target symptoms of worry and anxiety only for the three participants (Mrs. Liu, Mrs. Huang, and Mr. Lin) without comorbid depression. For these cases, RCIs computed for Ch-PSWQ and STICSA-Trait data were statistically significant (*p* < 0.05) at termination and 4mFU except in one case, indicating reliable improvement in pre-post scores. At the 4mFU, Ch-PSWQ scores were attenuated for Ms. Liu due to an acute stressor in the month prior to the assessment period (husband’s deportation proceedings; see Chang et al., 2016). The two participants (Mrs. Chan and Ms. Xiao) who did not show improvements in worry and anxiety were both older (in their 50s) and had comorbid depression.

Next, the clinical significance of symptom changes for the three improved cases was determined by comparing scores to normative data. Ch-PWSQ and STICSA-Trait scores moved from the dysfunctional range to the normal range of functioning (Behar et al., 2003; Van Dam et al., 2013) post-treatment and at the 4mFU for two and three participants, respectively. Clinically significant improvements on anxiety (STICSA-Trait) were observed by session 4 and maintained through the 4mFU, whereas improvements in worry (PWSQ) were more gradual.

#### Changes in Secondary Outcomes

By the end of treatment and through the 4mFU, depression scores dropped to negligible levels (PHQ-9 < 5) for the three patients with pure anxiety. For the two with comorbid MDD, one fell to the “mild” range of symptoms (5–9) whereas the other remained in the “moderate” range (10–14) through the 4mFU (Kroenke and Spitzer, 2002). Scores on the AAQ-II also dropped below the clinical range for the three patients with pure anxiety, suggesting improvements in cognitive flexibility and emotional avoidance (Bond et al., 2011). Finally, only two patients showed clinically significant improvements in interpersonal functioning and SR functioning (Mrs. Huang and Mrs. Chan, respectively).

### Subjective Experiences of Treatment: Patient and Therapist Perspectives

#### Patients

During the debriefing interviews, all but one patient (Ms. Xiao) described significant changes in anxiety and worry (see Table 2). To explore the effects of cultural shaping of the therapeutic relationship identified in Phase 2 of the adaptation process, we explored patients’ views of the relationship and implications for treatment process. Most patients described their relationship with the therapist as initially more formal and hierarchical, gradually shifting to a warmer, more collaborative dynamic over time. Mrs. Chan noted: “In the beginning I feel like I am seeing a medical doctor. Now…our relationship is like talking to a friend, a friend that I can talk to about things that are weighing on my mind.” Mrs. Liu described a similar shift,

At first, I would keep a lot of things in my mind, I would not tell [the clinician] about it. But I started to tell her about some things, and she listens to me very patiently. Then she would tell me that if I have these problems, what I can do to solve it. She teaches me from different aspects, I can do this, or I can do that to solve it. Then I can pick the most useful way for me. She will let me decide which way is most helpful for me. So I think this helps to improve our relationship. She will not be like, ‘Oh this is the only way, you have to do this.’ Maybe this idea works for other people, but it might not work for me. She gives me a lot of options.

As a whole, participants felt validated and accepted by their therapists. Mr. Lin reflected, “She’s very patient. I think this is very essential. I don’t think she tries to change me. She just want to help me to accept these things, how to apply it in my life, and how to change my attitude from different ways.” Study clinicians and patients were able to negotiate a working relationship in which patients were empowered to take an active role in their treatment and strengthen their coping capacities. Patients’ positive responses to this more patient-centered, collaborative approach to delivering TCT suggest the adaptability of the intervention to different cultural models of the therapeutic relationship.

Patients also provided feedback on the specific treatment components that they found to be most helpful. As a group, they felt that the Taoist principles were helpful, although each found a few principles to be more relevant to their life circumstances than others. One patient expressed a desire to learn more: “I think that eight principles are too little, there should be more. I feel like if you have eight then there should be more. If there’s an unexpected event, I wonder if there are more principles to use and help.” Several reported appreciating the handouts and consulting with them regularly, suggesting that this particular cultural adaptation was beneficial. Two participants reported that they could have benefitted from more time studying each principle to ensure deeper understanding before moving onto the next. Whereas several reported difficulties doing homework regularly, others found it very helpful [“I did all my homework. I think doing thought records is very helpful for me. Because when you are very stressed out, this is what (therapist) taught me, you write it down, and it will help you to relax and your thoughts won’t be so extreme. So for me, this is very helpful.”].

#### Clinicians

In reflecting on the three cases that she worked with (Mrs. Liu, Ms. Xiao, and Mr. Lin), the female clinician observed that the therapy was relatively easy to learn given her prior training and experience with CBT. However, she found it to be different in interesting ways:

It was kind of interesting because it incorporated these Taoist principles, which patients with a Chinese cultural background have heard of. All 3 of my patients have never studied these principles in depth but the principles were there in their cultural knowledge, so I think the cultural relevance of the treatment made it easier for them to accept. I think because it was culturally relevant, the patients have more confidence in the treatment and my guess is that it makes it more effective for them. In terms of the actual teaching content, both of us had to learn it at the same time, but for me [the Taoist principles] were the most interesting part of the treatment. It was easy to engage the patients in the discussion about the principles, not only because it comes from their culture but because also because there are new ways of thinking that are inherent in the principles so I think it was particularly engaging for the patients to think about what exactly they mean and how they could apply to their situation. Something outside of the patient’s typical framework of thinking about their problems. It’s new but at the same time, old and familiar because it comes from the culture.

However, the male clinician who worked with Mrs. Chan and Mrs. Huang cautioned that “just because the clinician and the patient are Chinese, doesn’t mean we don’t need to go through a detailed discussion about the principles.” Looking back on his work with Mrs. Chan, he realized that he made certain assumptions related to their shared identities as “older generation, Chinese American immigrants,” who would have “learned these kinds of principles from life experience, our parents taught them to us, in everyday life, or you learn from school. There was an underlying communication that “You already know it, I don’t need to go into detail to teach you these principles.” So the details were ignored. But the concepts can be diversely defined by myself or the client.” He goes on:

For example, if you hear carefully in the transcript—[Mrs. Chan] likes to use 順其自然 [Let nature take its course]. But she interpreted it differently and had to learn a new way of interpreting it. She used it in a very, very passive way—just follow what comes and never fight back. In her sense of the principle, it is a way of giving up. Actually, it means follow the flow to do what you are supposed to do—not against the flow because that will be useless. These subtle differences in interpretation played out in the session.

Here, the clinician emphasizes the importance of preserving the didactic components of the intervention – taking time to carefully instruct the patient in how the principles should be interpreted. This feedback was incorporated into the revised treatment manual (TCT v3) in the form of additional prompts for the clinician to assess the patients’ interpretation of each principle, thoroughly present the standard interpretation with numerous examples, and clarify any misunderstandings or conflicts in interpretation.

## Discussion

This study represents a rare attempt to adapt and manualize a promising therapy developed in China for dissemination in the United States context. In the absence of a complete training manual, we began by describing the procedures, techniques, and content of the original CTCP intervention through an iterative process of observation, transcription, and discussion of actual treatment cases. This bottom-up process allowed us to faithfully record how the intervention is typically delivered outside of a research setting. Guided by models of cultural adaptation and implementation science (Graham et al., 2006; Durlak and DuPre, 2008; Bernal et al., 2009; Hwang, 2009), we then consulted with subject-matter experts and bilingual/bicultural clinician stakeholders to evaluate the original protocol and identify potential barriers and enablers for successful implementation with Chinese immigrants with GAD. This process led to a number of changes aimed at improving clinicians’ self-efficacy to deliver the intervention (strengthening theoretical understanding and foundational knowledge about Taoism), standardize assessment, teaching, and application of core concepts (revising assessment procedures, providing teaching examples and resources), and provide resource supports to patients new to the therapy process and Taoist concepts (preparing patient handouts and forms).

Even with a small pilot sample of five participants, we gleaned a number of important insights that may be used to inform future research. From a feasibility perspective, we were successful in recruiting participants and retaining them for the duration of the treatment. However, we encountered a number of methodological challenges that would need to be addressed in subsequent trials. First, although all participants completed the treatment, barriers to attending sessions on a weekly basis stretched the expected treatment period of 4–5 to 8–12 months across the sample. More time between sessions made it difficult to cover the session agenda, as more time was needed for catch up and review. Second, participants were required not to alter any medications they were taking; however three participants independently stopped, decreased, and increased their medication, respectively, over the course of the study. This introduces a confound, which makes it difficult to isolate the effects of TCT. Although other treatment studies with non-Chinese samples document similar patient interfering behaviors (e.g., Roemer et al., 2008), our clinician stakeholders reported that these behaviors are particularly common in working-class Chinese immigrant patients, due to limited control over work schedules and looser attitudes toward medication management.

In an effort to study an externally valid sample in this exploratory investigation, we also included those who had comorbid conditions as long as they had a primary diagnosis of GAD. Including two cases with GAD and major depression revealed a stark difference in treatment outcomes (discussed below) that suggest the potential need to further refine the treatment model. Nevertheless, this variability within the sample introduces threats to internal validity that would need to be considered in future studies.

With regard to the acceptability of TCT, we expected that participants might find it to be culturally familiar (and therefore highly credible); however, ratings of treatment credibility and expectations were in the moderate range. Participants did not report familiarity with formal Taoist concepts nor did they assume it would be helpful as a mental health treatment. However by termination, they rated the Taoist concepts as important for promoting health and well-being. Research is needed to determine how acceptability ratings compare between TCT and non-adapted cognitive approaches to treating anxiety, and whether these moderate initial ratings reflect skepticism about mental health treatment in general (Sue et al., 2012), or TCT in particular.

While we recognize the limitations of a study with few participants and without a control group, results provide preliminary evidence that TCT may be successfully implemented in the United States context. Our findings mirror previous investigations reporting CTCP to be effective in significantly reducing symptoms of generalized anxiety (Zhang et al., 2002) in China. All participants with “pure” anxiety showed statistically and clinically significant reductions in anxiety and worry, psychological inflexibility, and emotional avoidance, moving them from the dysfunctional to the normal range by termination. However despite other studies showing that CTCP has been successful in treating depression in China (Li et al., 2014; Liu and Yu, 2015), the two participants with comorbid depression reported few improvements. In reviewing the findings with the study clinicians, we wondered whether the Taoist emphasis on 無為 (*wuwei*), sometimes translated as “actionless action” or “non-doing,” may have promoted a sense of helplessness and hopelessness among those vulnerable to depression. As illustrated in Mrs. Chan’s case, she interpreted 無為 to mean a passive acceptance of life’s difficulties, rather than a more active approach to dealing with things as they are, which may include patiently observing the situation to determine the best course of action. These pilot findings suggest the importance of monitoring patients’ understanding of the Taoist principles and possibly considering other treatment modifications to specifically address the low self-efficacy and fatalism expressed among those with comorbid depression.

In conclusion, this small pilot trial suggests that TCT can be successfully implemented with Chinese immigrant patients receiving services from bilingual/bicultural clinicians with little formal background in Taoism. Results suggest that Taoist principles of acceptance and flexible adaptation to natural laws may be helpful to Chinese immigrants coping with anxiety and worry. Findings should be replicated in a randomized controlled trail with a much larger sample and compared with treatment as usual or an unadapted CBT to assess the specificity and generalizability of these results.

## Data Availability Statement

The raw data supporting the conclusions of this article will be made available by the authors, without undue reservation.

## Ethics Statement

The studies involving human participants were reviewed and approved by New School University Human Research Protection Program. The patients/participants provided their written informed consent to participate in this study.

## Author Contributions

DC was the principal investigator of this project and was responsible for all phases of the research including developing the treatment manual, training and supervising the study clinicians, overseeing participant recruitment, data collection and analysis, and drafting the manuscript. NN was the project manager, overseeing participant recruitment and data collection, and conducting the data analysis. TC and TH were key clinician stakeholders and served as study clinicians for these pilot cases. IM was a research assistant who assisted with data management. YC and YZ assisted with recruiting and recording the cases collected in Beijing and Shanghai and provided technical assistance in clarifying the intervention components and techniques. YZ was one of the original developers of CTCP. All authors contributed to the article and approved the submitted version.

### Conflict of Interest

The authors declare that the research was conducted in the absence of any commercial or financial relationships that could be construed as a potential conflict of interest.

The reviewer WT declared a shared affiliation with one of the authors, DC, to the handling editor at time of review.
